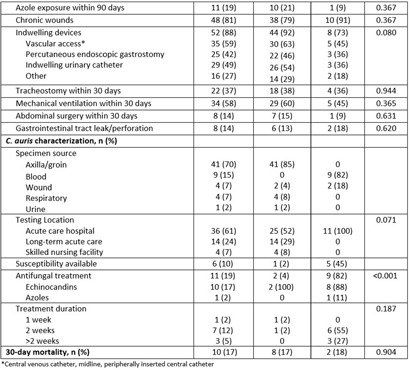# Risk Factors and Outcomes of Candida auris in Southeast Michigan

**DOI:** 10.1017/ash.2024.234

**Published:** 2024-09-16

**Authors:** Ambreen Malik, Anita Shallal, George Alangaden, Geehan Suleyman

**Affiliations:** Henry Ford Hospital; Henry Ford Health System and Wayne State Univ

## Abstract

**Background:** Candida auris is an emerging multidrug resistant fungus that presents a serious global health threat and causes severe infections with a high mortality rate in hospitalized patients with significant underlying comorbidities. We describe the risk factors and clinical outcomes associated with C. auris in Southeast Michigan. **Methods:** This is a retrospective case series of culture-positive C. auris patients who had contact with our healthcare facility in Detroit from 2021 to 2023. We evaluated demographics, comorbidities, risk factors, and outcomes. A comparative analysis of colonized and infected patients was performed. **Results:** Forty-eight (81%) colonized and 11 (19%) infected patients were included (Table); 70% were male with median age of 66 years. All variables were comparable between the two groups except chronic kidney disease, which was significantly more prevalent among colonized patients (40% vs 0, p=0.011). All patients had prior exposure to acute care hospital (ACH), 37% to long-term acute care hospital, and 42% to skilled nursing facility within 1 year of diagnosis. Chronic wounds, prior broad-spectrum antibiotic use, and indwelling devices were prevalent in both groups; more than half required mechanical ventilation in the last month, and one third had tracheostomy at the time of C. auris detection. Almost 60% had a prior history of drug-resistant organisms, including multi-drug resistant gram negative (37%) and carbapenem-resistant (20%) organisms. Blood (82%) and wound (18%) were sources of invasive candidiasis. More than half (61%) of the testing was performed at ACH. Nine patients (82%) with invasive disease were treated with echinocandins (88%); among the colonized, two (4%) were treated with echinocandins but had persistent colonization. Thirty-day mortality was not significantly different among the two groups and was nearly 20%. **Conclusions:** In this large cohort study, a history of healthcare exposure, drug-resistant organisms, use of broad-spectrum antibiotics, indwelling devices, and chronic wounds were common risk factors among C. auris patients. Limiting the use of broad-spectrum antimicrobials and invasive devices, adherence to infection prevention and control practices, and interfacility transfer communication are important mitigating strategies to reduce the incidence and spread of C. auris.

**Figure:**
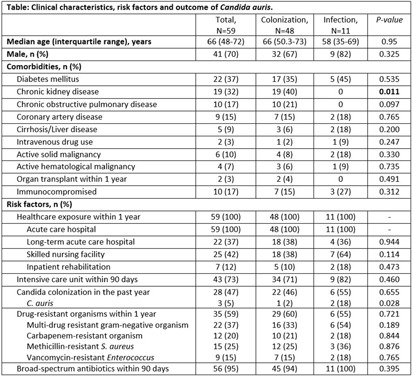

**Figure:**